# HPV vaccine narratives on Twitter during the COVID-19 pandemic: a social network, thematic, and sentiment analysis

**DOI:** 10.1186/s12889-023-15615-w

**Published:** 2023-04-14

**Authors:** Jean-Christophe Boucher, So Youn Kim, Geneviève Jessiman-Perreault, Jack Edwards, Henry Smith, Nicole Frenette, Abbas Badami, Lisa Allen Scott

**Affiliations:** 1 School of Public Policy, University of Calgary, 906 8th Avenue S.W. 5th Floor, Calgary, AB T2P 1H9 Canada; 2 Provincial Population and Public Health, Alberta Health Services, Holy Cross Centre, 2210 2 St SW, Calgary, AB T2S 3C3 Canada

**Keywords:** HPV vaccine, Twitter, Social media, Social Network Analysis, COVID-19

## Abstract

**Introduction:**

The COVID-19 pandemic has increased online interactions and the spread of misinformation. Some researchers anticipate benefits stemming from improved public awareness of the value of vaccines while others worry concerns around vaccine development and public health mandates may have damaged public trust. There is a need to understand whether the COVID-19 pandemic, vaccine development, and vaccine mandates have influenced HPV vaccine attitudes and sentiments to inform health communication strategies.

**Methods:**

We collected 596,987 global English-language tweets from January 2019-May 2021 using Twitter’s Academic Research Product track. We determined vaccine confident and hesitant networks discussing HPV immunization using social network analysis. Then, we used a neural network approach to natural language processing to measure narratives and sentiment pertaining to HPV immunization.

**Results:**

Most of the tweets in the vaccine hesitant network were negative in tone (54.9%) and focused on safety concerns surrounding the HPV vaccine while most of the tweets in the vaccine confident network were neutral (51.6%) and emphasized the health benefits of vaccination. Growth in negative sentiment among the vaccine hesitant network corresponded with legislative efforts in the State of New York to mandate HPV vaccination for public school students in 2019 and the WHO declaration of COVID-19 as a Global Health Emergency in 2020. In the vaccine confident network, the number of tweets concerning the HPV vaccine decreased during the COVID-19 pandemic but in both vaccine hesitant and confident networks, the sentiments, and themes of tweets about HPV vaccine were unchanged.

**Conclusions:**

Although we did not observe a difference in narratives or sentiments surrounding the HPV vaccine during the COVID-19 pandemic, we observed a decreased focus on the HPV vaccine among vaccine confident groups. As routine vaccine catch-up programs restart, there is a need to invest in health communication online to raise awareness about the benefits and safety of the HPV vaccine.

## Background

Human papillomavirus (HPV) is the most prevalent sexually transmitted infection (STI) in the world [1] and is associated with the development of multiple cancers (e.g., cervical cancer, anal cancer, oropharyngeal cancer) and health conditions (e.g., genital warts) [2]. Most of these cancer cases are caused by nine types of HPV [2], and these high-risk HPV types are preventable with a safe and effective HPV vaccine that has been available since 2006 [3]. Furthermore, HPV is attributable to 4.5% of all cancers (8.6% in women and 0.8% in men) [2]. To reduce the global burden of cancer, and particularly cervical cancer, the World Health Organization (WHO) has set a goal that by 2030, 90% of girls in the world will have received the HPV vaccine by age 15 [4]. Yet globally, we are still not on track to meet this goal, even in high-income countries with consistent access to the HPV vaccine. Researchers have theorized that in high-income countries, misinformation plays a large role in vaccine hesitancy and, as a result, a sub-optimal HPV vaccination rate [5].

The increasing use of online sources for health information among the public [6–7] also has the potential to impact vaccination uptake. While the online environment has the potential to enhance knowledge about vaccines and improve attitudes towards immunization as people share knowledge and experiences, it can also create an environment that spreads and amplifies misinformation about vaccination, including the HPV vaccine. In their study of HPV related tweets, Dunn and colleagues [8] found that approximately 25% of tweets espoused negative sentiments towards HPV immunization, and that exposure to these messages increased the likelihood of the reader subsequently posting their own negative-sentiment tweets towards the HPV vaccine. After critical appraisal, the researchers found that negative-sentiment tweets on HPV vaccination tended to be characterized by misinformation, and often leveraged opinion or anecdotes as evidence, rather than citing scientific information [8]. When these negative messages are shared in communities formed on social media platforms such as Twitter, they may be widely spread and rapidly amplified as they reverberate through social networks, leading to the pervasive spread of “unbalanced, distorted, or inaccurate information about vaccines” [9, p.2] that becomes difficult to counter with health promotion messaging.

Exposure to misinformation, or false information, has emerged as a public health concern since research has shown that even small exposures to anti-vaccination messaging in online settings (even as little as five minutes), can have a measurable negative impact on individuals’ attitudes and intent towards immunization [10–13]. These exposures to negative messaging can have measurable impacts of vaccination rates, as demonstrated by a large American study linking state-level HPV vaccine rates to the predominant tone on social media [14]. On social media, sentiment towards HPV vaccination varies by platform, with Twitter [15–17] and Instagram [18] tending to be more positive toward HPV vaccination, whereas YouTube [19] and Facebook [20] being more negative. Unfortunately, research has shown that users exposed to HPV vaccine messages are more likely to remember the messages surrounding alleged harms of the vaccine rather than its potential benefits [21]. This supports research suggesting public health strategies which emphasize the provision of statistical information to vaccine sceptics can be less effective than information which focuses on conveying general takeaways and is framed to have an emotive appeal to the target’s personal beliefs and reference group [22–23]. Emotional resonance of information is highly impactful; one study demonstrated that parents who are exposed to both positive and negative messages about the HPV vaccine were less likely to vaccinate their children compared to those only exposed to positive messaging [24]. Overall, the literature demonstrates that acceptance and uptake of the HPV vaccine is strongly tied to the information the prospective recipient is exposed to, with misinformation driving negative sentiment negatively impacting the recipient’s likelihood of consenting to vaccination.

The past two years of the Coronavirus Disease 2019 (COVID-19) pandemic have seen increases in online interactions during periods of isolation and social distancing [25], as well as the intensification of the spread of health misinformation online [26–27]. While discussions around COVID-19 have increased exponentially, recent research tracking public conversations has indicated that alongside increases in discussions of COVID-19 and COVID-19 vaccination, interest in other vaccines has not decreased and in some periods has, in fact, increased [28–29]. Some researchers have hypothesized this is due to benefits stemming from improved public awareness of the value of vaccines [30], while others worry that the rapid development and approval COVID-19 vaccines and subsequent vaccine mandates may have damaged public trust in institutions and impacted acceptance of other vaccines [31]. This includes the HPV vaccine, which has experienced parental mistrust due to the perception that the vaccine is too new [32–33].

This raises the question of whether the COVID-19 pandemic had on impact on vaccine hesitancy generally. Did greater public awareness of vaccines as a result of the saturated information environment caused by the COVID-19 pandemic increase vaccine scepticism in the public? This paper seeks to examine how discussions on the COVID-19 vaccines shaped the public’s attitude toward HPV vaccination. While the existing work on vaccine hesitancy largely suggests vaccine sceptics form their opinions about vaccines *a priori* to considering new vaccines, this research was done on novel vaccine development for smaller scale public health emergencies [34].

Moreover, the COVID-19 pandemic has influenced routine immunization programs through delays to childhood immunization and school- and community-based immunization programs [35–37]. Combined with decreases in screening uptake in community health centres due to the COVID-19 pandemic, researchers are expecting to see rises in vaccine preventable diseases [38] and cancer incidence [39–40]. Therefore, in this context, there is an urgent need to understand how the COVID-19 pandemic has impacted attitudes and sentiments on the HPV vaccine to inform the development of health communication strategies that address misinformation, with the goal of increasing vaccine acceptance and encouraging HPV vaccine uptake.

### Objectives

1. To describe and characterize vaccine hesitant and vaccine confident networks of tweets about HPV and HPV vaccination on Twitter from January 2019 to May 2021.
2. To determine how HPV vaccine themes and sentiments differ between vaccine confident and vaccine hesitant networks.
3. To determine whether the themes and sentiments towards the HPV vaccine changed during the COVID-19 pandemic and the COVID-19 vaccine rollout.

## Methods

### Data collection

The Academic Research Product Track Application Programming Interface (API) from Twitter was used to collect global tweets from January 2019 to May 2021. Keywords related to HPV vaccination were informed by a rapid review of 13 peer-reviewed articles published between 2015 and 2020 focused on HPV vaccination. These keywords (e.g., “HPV” OR “Gardasil” OR “Cervarix”) were used to gather tweets and re-tweets on HPV vaccination from individual Twitter accounts. From this same dataset, a Boolean search using the keywords (“COVID” OR “corona”) was conducted to collect conversations around the COVID-19 pandemic and vaccinations. All data was imported, cleaned, and analyzed in Python version 3.8.5.

### Social network analysis (SNA)

**Fig. 1:**
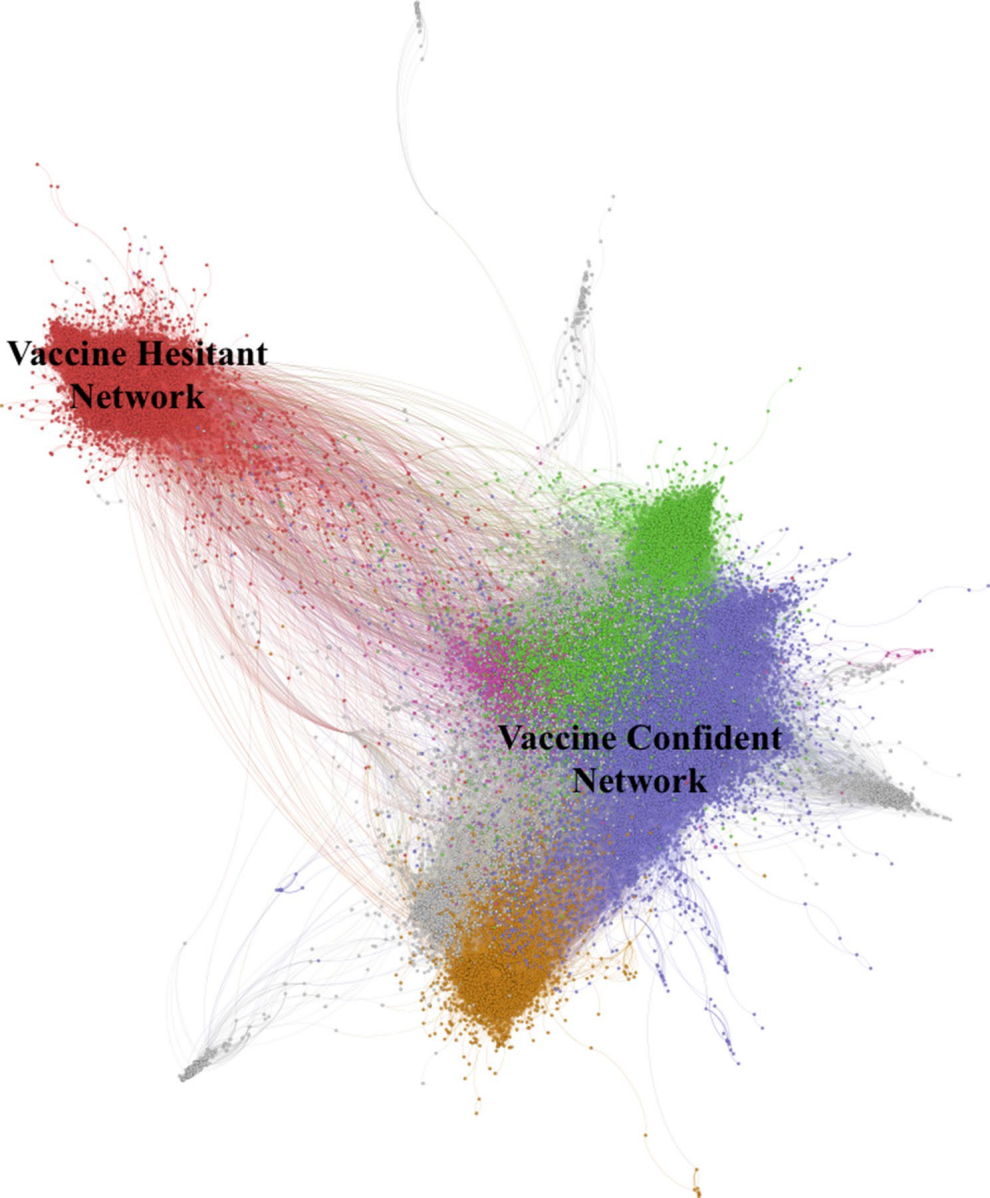
Full network of HPV Immunization Tweets

For this study, we first used SNA to identify social media accounts expressing confidence in or hesitance toward HPV vaccination. We created a network displaying the relationship between user accounts and retweets of other accounts (Fig. 1). This was to identify influential accounts, their level of influence, and their connections. While we recognize tweets do not provide an exact indication of like-mindedness, on aggregate, users who exhibit social or intellectual homophily are more likely to interact with each other on social media [42]. The Louvain modularity method was used to determine subclusters of online communities discussing HPV vaccination [43]. The key influencers from the subclusters were studied to classify vaccine confidence and hesitancy networks in the social media space.

### Sentiment analysis and thematic clustering

Social media conversations around HPV vaccines were analyzed using natural language processing. The tweets were first cleaned and processed for analysis using the Natural Language Toolkit (NLTK) library in Python. The topic themes of the tweets were identified using a mixed-method approach of unsupervised machine learning and qualitative content analysis of vaccine confident and vaccine hesitant tweets. An agglomerative hierarchical cluster model was first developed to detect clusters of topic themes [44]. We then measured the term frequency-inverse document frequency (TF-IDF) of the clusters which calculates the relevance of a word (term frequency) in a document among a collection of documents (inverse document frequency). This was used to measure undervalue words that appeared often and provided little information and overvalue words that appeared only occasionally in the corpus, but often in some documents. We performed qualitative content analysis to infer themes from our clustering model, using both TF-IDF outputs and a typology of themes that included examples and definitions of each theme. This allowed us to identify the predominant narrative theme in each cluster. The cluster analyses were conducted independently by two analysts (SK and JE), and the theme labelling of the clusters was reviewed to ensure consistency in the coding process. The review process was repeated until the consensus of the topic theme for each cluster was reached.

To determine sentiment, we used a supervised model where a random sample of tweets were labeled by our analysts along three categories: positive, negative, and neutral. We employed a BERT (Bidirectional Encoder Representations from Transformers) [45] to classify the sentiment of the tweets. BERT was used to learn the contextual relationship between words and to generate word embedding features by converting each tweet into a 768-dimension vector. The model was further fine-tuned by adding a sentiment classification layer to classify whether a tweet is negative, neutral, or positive in tone. The supervised model to classify sentiments for vaccine confident tweets had an accuracy score of 96.8% and the vaccine hesitant supervised model had an accuracy of 97.3%.

## Results

An HPV Twitter dataset was collected from January 2019 to May 2021 consisting of 596,987 tweets from 316,835 individual Twitter accounts. From this data, we used SNA to detect the polarization of vaccine hesitant and vaccine confident conversations around HPV disease and immunizations. Figure 1 displays the social network of Twitter accounts and retweet clusters of HPV-related discussions among the vaccine hesitant and vaccine confident communities. In total, 95,908 tweets (16.1%) were clearly associated with vaccine hesitant networks in red, and 234,015 (39.2%) tweets were vaccine confident conversations.

### Sentiment analysis

**Fig. 2:**
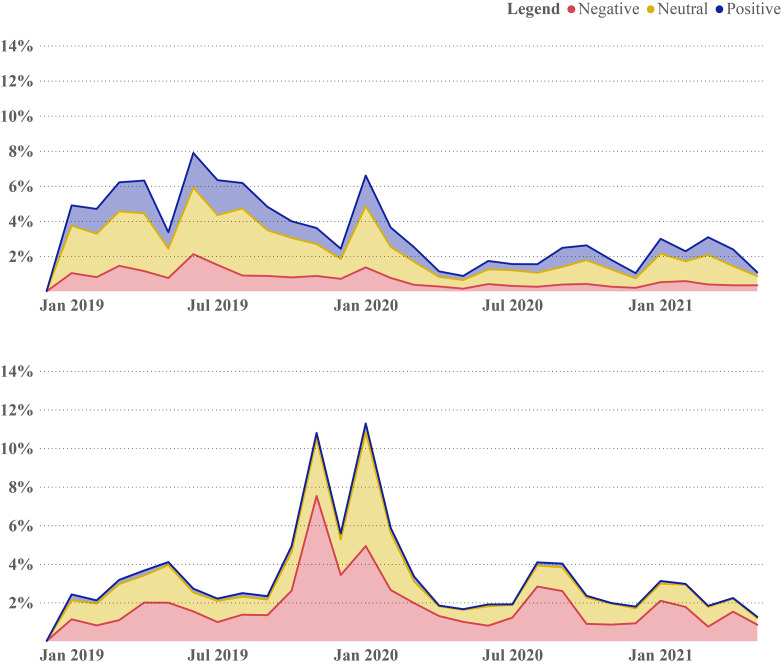
HPV Sentiment Analysis by Network Group Over Time

Figure 2 presents the distribution of sentiment in tweets over time by vaccine community type. The fine-tuned BERT model was trained to classify positive, neutral, and negative sentiment tweets. Our model identified 4,555 (4.7%) positive, 38,713 (40.4%) neutral, and 52,640 (54.9%) negative tweets from the vaccine hesitant community. The vaccine confident community produced 65,838 (28.1%) positive, 120,704 (51.6%) neutral, and 52,640 (22.5%) negative tweets. The overall proportion of all three sentiments increased in both vaccine hesitant and vaccine confident groups around February 2020, when the WHO declared COVID-19 outbreak a Public Health Emergency of International Concern. Comparing the sentiment of tweets in both vaccine confident and vaccine hesitant groups, as expected, the tweets of vaccine hesitant individuals were overall more negative in tone than those who were vaccine confident.

### Thematic clustering: vaccine safety, efficacy, and mistrust in institutions

Through unsupervised machine learning, we identified the key narratives driving HPV vaccine hesitancy and confidence on social media. Tables 1 and 2 show the results from our topic theme analysis and utilization of TF-IDF output.

**Table 1:** Occurrence of HPV Immunization Themes by Vaccine Hesitant Networks

Topic	Topic Keywords Bi-gram	Themes	Tweets (n)
1	dead bathroom, bathroom floor, serious nervous, kills people, vaccine caused, neurotoxic aluminum, including neurotoxic, allegedly injured, filed lawsuit, healthy teenager, basketball health, went downhill, watch daughter, suffer everyday, daughter suffer, companys dangerous, facial swell, seizurelike episode, anxiety american, rises depression, autions adverse, harms cautions, contradictions questionable, gardasil science	Vaccine Safety	60,436
2	rise cervical, research uk, cancer research, generation receive, cancer rates	Vaccine Effectiveness	13,830
3	shot dr, testify hearing, hearing mandating, trick pharma, trial trick, mandate terrible, believe government, government trying, cannot believe, vaccine minors, passed bills	Mistrust Institutions/Elites	9,081

**Table 2:** Occurrence of HPV Immunization Themes by Vaccine Confident Networks

Topic	Topic Keywords Bi-gram	Theme	Tweets (n)
1	cases throat, diagnosed annually, cancer diagnosed, transient infection, infection afflicts, afflicts adults, elimination cervical cancer, hopes cervical, cervical cancer cured using, threats womens, worldwide cervical cancer, mother left, behind boys, drop cervical, vaccine linked, dramatic drop, dramatic cervical, contact enough, std skin to skin, genital war, get std,	Health Outcomes	89,964
2	advocate working, world cancer day release, hpv advocate, people sweden, laura brennan, campaigner laura, hospital thanks	Vaccination Campaign	57,504
3	huge news, vaccination introduced, stunning years, fall rates, reducing human, programmes substantial, prevents types, protect cancers, protecting adolescents, prevents death, pretty amazing	Vaccine Effectiveness	38,606
4	clogging internet, truth jab, bullshit, phony	Mistrust Towards Anti Vaxxers	26,717
5	rwanda could, wipe cervical, first country, could first, nigeria, kenyan parents please, offered free, september boys, boys school, vaccine first, boys prevent, thousands cancers	Vaccine Access	18,378

As depicted in Table 1, vaccine hesitant narratives fall into three broad themes. *Vaccine Safety* was a large theme of discussion accounting for approximately 64.1% (n = 60,436) of vaccine hesitant tweets. Vaccine safety tweets primarily focused on the side effects of the Gardasil vaccine and listed allegedly harmful compounds in the vaccine. The second topic centred around *Vaccine Effectiveness* (21.3%, n = 13,830), questioning the effectiveness of the vaccine in preventing cancer and sharing statistics of cervical cancer cases in the UK to support this argument. Finally, approximately 14.7% (n = 9,081) of the vaccine hesitant network expressed *Mistrust in Institutions* and showed resentment towards the US government mandating HPV vaccines in school.

Conversely, the vaccine confident network discussions were framed in five distinct ways, as shown in Table 2. *Health Outcomes* were largely emphasized by the vaccine confident, making up around 38.4% (n = 89,964) of conversations. The narratives of this topic mainly focused on the impacts of the virus. These tweets emphasized HPV-related diseases such as head, neck and oropharyngeal cancer, awareness of cervical cancer in women, and sexually transmitted infections. The second predominant theme was *Vaccination Campaigns* (24.6%, n = 57,504), which highlighted the work of public health organizations and HPV advocates and promoted World Cancer Day or HPV-related social events. *Vaccine Effectiveness* (16.5%, n = 38,606) was the third most prevalent theme in the vaccine confident space, and saw tweets shared focusing on the effectiveness of the vaccine in eradicating cancer-causing HPV infections. Another topic theme expressed *Mistrust Towards Anti-vaxxers* (11.4%, n = 26,717) spreading HPV vaccine misinformation and called out myths and conspiracy theories that are shared by anti-vaxxers. Finally, *Vaccine Access* (7.9%, n = 18,378), related primarily to the procurement and distribution of HPV vaccines, was the last theme observed in the vaccine confident space. This topic also focused on the importance of HPV vaccines for males and supported HPV vaccination programs for boys in schools. Furthermore, vaccine access tweets highlighted the free distribution of HPV vaccines in Kenya and acknowledged the prospective long-term reductions in cervical cancer in Rwanda due to the implementation of its HPV vaccine program.

**Fig. 3:**
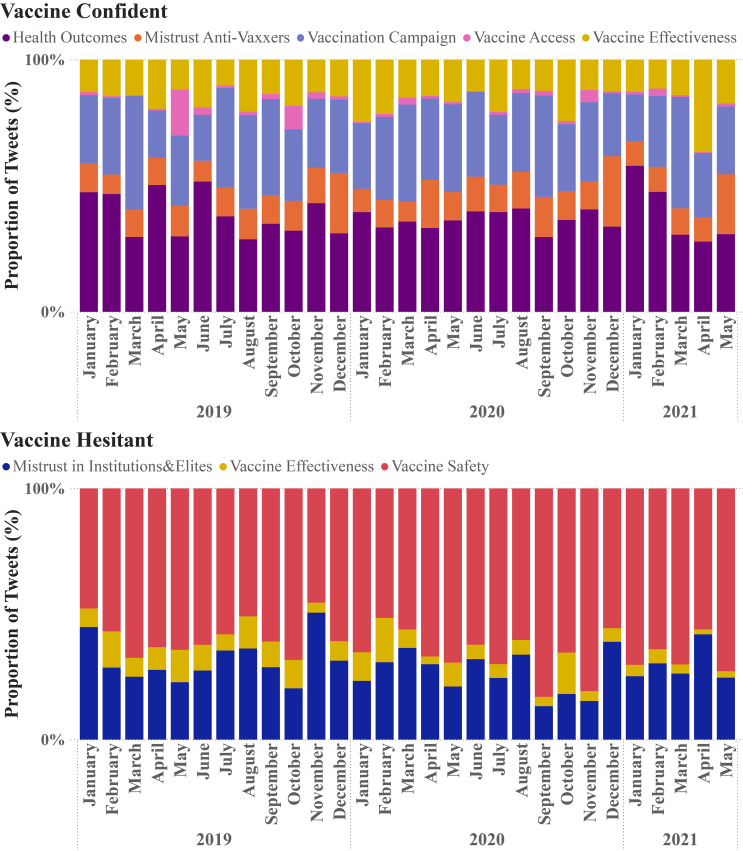
Occurrence of HPV Immunization Themes by Vaccine Confident and Vaccine Hesitant Networks

Figure 3 displays the proportion of vaccine confident and vaccine hesitant topic themes from January 2019 to May 2021. Health outcomes remained the primary topic of discussion and vaccination campaigns were the second most common theme in vaccine confident discussions. The long-term trends in the data show vaccine safety as the predominant theme of discussion among the vaccine hesitant cluster.

### Co-discussion of COVID-19 and HPV in online conversations

**Fig. 4:**
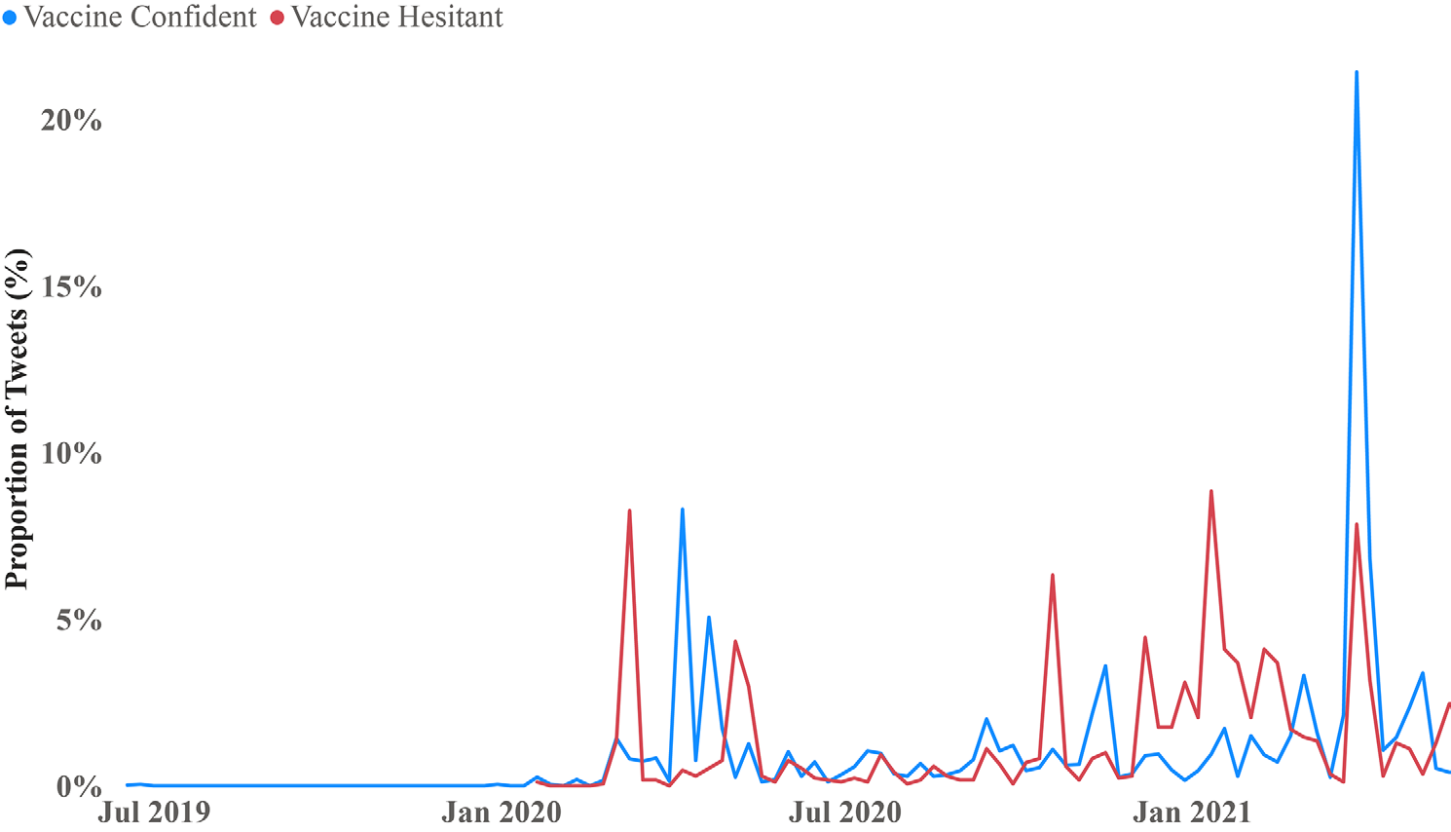
Distribution of COVID-19 mentions by HPV Vaccine Confident and Vaccine Hesitant Networks

The trends of COVID-19 mentions in tweets for HPV vaccine confident and vaccine hesitant networks are illustrated in Fig. 4. Vaccine hesitant tweets mentioning COVID-19 picked up in March and June of 2020, before steadily fluctuating from October 2020 to April 2021. In contrast, the proportion of tweets mentioning COVID-19 among vaccine confident discussions showed a slight peak in April 2020 before a significant peak in April 2021. Both vaccine confident and hesitant networks showed a lull of COVID-19 mentions in their tweets over the summer and early autumn of 2020.

### Changes in interactions of vaccine confident and vaccine hesitant networks

**Fig. 5:**
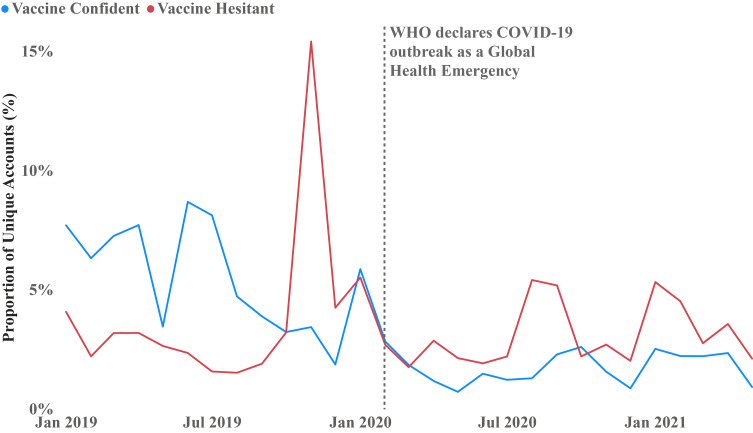
Distribution of Vaccine Confident and Vaccine Hesitant Unique Twitter Accounts

Between January 2019 to May 2021, we identified 93,498 unique Twitter accounts expressing vaccine confidence and 234,015 expressing vaccine hesitancy. Figure 5 displays the new unique accounts that tweeted HPV-related tweets that month. Notably, following the WHO’s declaration of COVID-19 as a Public Health Emergency of International Concern, the number of new vaccine confident Twitter accounts decreased, while the number of new vaccine hesitant accounts increased.

## Discussion

Using data from over 500,000 tweets, we conducted social network analysis, sentiment analysis and thematic clustering to visualize the HPV vaccine hesitant and vaccine confident networks and describe the sentiments and themes of these networks’ conversations. Further, we assessed whether there had been any changes to sentiments or themes in each network during the pandemic and subsequent COVID-19 vaccine rollout. While similar studies have examined the online conversation regarding the HPV vaccine using social network analysis and machine learning techniques [9, 41, 46–48], the present study contributes to the body of research by examining the influence of the COVID-19 pandemic on HPV vaccine sentiments in vaccine confident and vaccine hesitant networks on Twitter. Thus, this study provides a novel context with which to conceptualize the online discourse around HPV vaccination.

### Characterisation of vaccine confident and vaccine hesitant network

Looking at the aggregate of the collected tweets, certain characteristics are particularly prominent. First, the HPV vaccine hesitant community is small and tightly clustered, comprising a homogenous and densely connected community facilitating the flow of information and socialization of its members to group norms [49–50]. In contrast, the HPV vaccine confident community is larger, but also not as tightly grouped, consisting of three distinct and somewhat separated sub-communities. Both communities can be seen in Fig. 1 and are highly polarised, with few connections existing between them. This also reflects the limited extent to which information can traverse between the communities and indicates that most of the information that users in each community are exposed to originates from within their own community. While this has the effect of insulating the vaccine confident accounts from potential misinformation originating from the vaccine hesitant space, it also means that there are limited opportunities for organic exchange of ideas to occur between networks. Thus, in this network structure, it is less likely that accurate information will be disseminated to the vaccine hesitant space from vaccine confident influencers. Our results also indicate that there are few individuals or groups who act as bridges between these polarized groups. Therefore, from a public health perspective, there is a need to use other strategies to reach vaccine hesitant groups, and much of the current recommendations have focused on training public health experts or health professionals to address misinformation in online forums using plain-language communication strategies [51–52]. Yet, more research is still needed on how to effectively bridge the communication divide between vaccine confident and vaccine hesitant groups.

### Sentiment and thematic clustering

The types of sentiments expressed by the two communities exhibit substantial variance. As shown in Fig. 2, the HPV vaccine hesitant discourse is largely composed of negative sentiments. This appears to stem from the vaccine hesitant community’s inherently unfavourable view of vaccination, be it regarding the safety of the vaccine itself or the intentions of those promoting it. In contrast, the HPV vaccine confident discourse primarily consists of neutral sentiments. This may be a result of this community often engaging in more dispassionate discussions about empirical evidence and academic studies surrounding the vaccine and its outcomes, disseminating media coverage of related stories without offering comment of their own, or simply sharing scientific evidence and information without adding any specific message or interpretation.

With respect to thematic clustering, there were both similarities and differences between the vaccine hesitant and confident communities. First, prominent themes in both communities (vaccine safety for the hesitant and health outcomes for the confident) are concerned with the potential health effects or consequences associated with receiving the HPV vaccine. Vaccine efficacy, for its part, remains marginal as a thematic cluster, suggesting that neither group emphasizes, on aggregate, the specific protection against HPV provided by the vaccine, and that focus is, instead, put on other health benefits or side effects. Furthermore, both communities also express mistrust in groups promoting the opposing narratives toward the HPV vaccine, i.e., institutions/elites or anti-vaxxers, again highlighting the deep polarization between these two communities. The predominance of concerns regarding vaccine safety and tweets demonstrating a mistrust in institutions and societal elites reflect similar topics to those prevalent on anti-vaccine websites [53].

HPV vaccine hesitant and confident communities also significantly differ in other manners and the discussion of consequences of HPV vaccination within the hesitant group tends to focus on perceptions the vaccine is unsafe. A large amount of discourse is focused on Merck, purporting that their studies contain contradictions, that the science backing up their claims is unsound, and highlighting ongoing lawsuits involving the Merck-produced Gardasil vaccine. In addition, members of the hesitant community frequently share stories about specific cases of adverse or lethal reactions to the HPV vaccine. In this respect, there is a greater focus on individual stories and anecdotes as opposed to sharing studies on a group of statistically significant size. There is also discussion about both acute and chronic side effects of the Gardasil vaccine, including swelling, epilepsy-like conditions, and negative mental health outcomes. Blame for these side effects is often placed on supposedly harmful chemicals or compounds found within the vaccine, including aluminum. Overall, the discourse surrounding the HPV vaccine in the vaccine hesitant community reflects concerns with severe side effects and the potential of the vaccine to harm recipients.

In contrast, within the vaccine confident group, discussions on consequences of HPV vaccination tends to focus on the benefits of the vaccine. They point to positive outcomes for women’s health, improvements to sexual health because of the uptake of a vaccine for a common STI, and general positive outcomes associated with receiving it. There is also a larger focus in this community on studies on the health effects of larger groups or populations, with proponents sharing studies conducted in jurisdictions where the HPV vaccine is widely available. Such studies indicate a decrease in the rates of cervical, throat, and anal cancers associated with HPV infections since the vaccine has become available. These studies further express hope that increased uptake of the vaccine could virtually eliminate several forms of cancer in the near future. Accordingly, vaccine confident individuals are likely to be positive about the benefits associated with the HPV vaccine.

Within the vaccine confident community, there is also discussion around vaccination campaigns to encourage people to get the HPV vaccine. World Cancer Day is often mentioned as an example of an opportunity to advocate for greater global levels of vaccine uptake. Discussions of offering the vaccine for free to boys, in addition to girls, are also present, pointing to increased effectiveness of the vaccine with greater population uptake, as well as positive health outcomes for males. The Irish HPV vaccine advocate Laura Brennan is also mentioned often in the vaccine confident network. Hers is one of the few individual stories popular amongst the vaccine confident community, surrounding her campaign for increased HPV vaccination after personally receiving a terminal cervical cancer diagnosis in 2017 [54].

There is, additionally, a negative discussion amongst the vaccine confident community surrounding the lack of access to the HPV vaccine due to global vaccine shortages for certain populations, namely in developing countries. Authors have highlighted issues around global equity as developed nations expand their vaccination programs to boys while developing nations do not have enough vaccine supply to vaccinate girls [55]. Within this discussion of global vaccine equity, there are success stories shared. For example, Kenya is framed as a nation that has implemented free vaccine distribution with exhortations to Kenyan parents to take advantage of this access [56]. Rwanda and Nigeria are often mentioned as nations that are capable of following Kenya’s lead in providing free vaccine access. Among the vaccine confident, these examples reflect the potential to combat cervical cancer globally, if vaccine supply meets demand.

Mistrust from both communities also exists as a theme, but such mistrust is targeted at different groups. Vaccine hesitant groups tend to mistrust institutions and elites, calling into question their motivations and intentions behind encouraging or mandating vaccination [57]. As such, if individuals believe these groups are incompetent or malicious, they may not trust that the stages of vaccine development have been carried out appropriately. Conversely, vaccine confident individuals tend to mistrust anti-vaxxers, accusing them of being ignorant and knowingly or unknowingly spreading misinformation [58]. Vaccine confident individuals are more likely to believe that the development of these vaccines has been carried out safely and competently, so may hold those opposed to what they perceive as a potentially lifesaving vaccine on a large scale in poor regard. They may also be more likely to believe that prominent anti-vaxxers have an inherent malicious or selfish motivation and that followers of them are ignorant or misguided. Both these themes concern the perceived spread of wrong or misleading information. Additionally, within the hesitant community, discussions of vaccine efficacy focus on a purported rise in cervical cancer rates amongst the vaccinated. Within the confident community, the exact opposite is observed in discussions surrounding declines in HPV infections and cervical cancer rates associated with receipt of the HPV vaccine. Yet again, this dichotomy in opinion between the two communities reflects two contrasting realities prevalent in the discourse surrounding the HPV vaccine.

### Effects of COVID-19 and COVID-19 vaccine rollout

Finally, an examination of the changes in sentiments and themes in Figs. 2 and 3, it can be observed what effects the outbreak of COVID-19 and the early stages of COVID-19 vaccine rollout had on the HPV vaccine confident and hesitant communities.

In Fig. 2, the HPV vaccine hesitant community shows a large uptick in discussion in late 2019 and early 2020, especially in negative sentiment discussions. This corresponds with legislative efforts in New York State to mandate the HPV vaccine for public school students [59], as well as the WHO declaration of COVID-19 as a Global Health Emergency [60]. By April and May of 2020, the discussions return to levels like those found earlier in 2019. COVID-19, up until May 2021, appeared to have had little aggregated effect on the amount or sentiment of discussion in the HPV vaccine hesitant community on Twitter. This may indicate that the vaccine confident community had temporarily turned their attention away from HPV and were more focused on the ongoing COVID-19 pandemic as COVID-19 became the predominant focus in public health. Today, as a result, as routine vaccination catch-up programs are increasingly implemented, there will likely be a need to invest in health communication efforts to increase awareness about the HPV vaccine and its benefits as public interest and discussion have not been at the forefront of the minds of members of the public, even those individuals positively disposed to HPV vaccines, throughout the pandemic. Future work should consider measuring how the COVID-19 pandemic decreased uptake for vaccination in general and HPV in particular.

The prevalence of HPV vaccine themes within the wider discourse, shown in Fig. 3, shows little change over the course of the COVID-19 pandemic during the time period studied. It seems that the pandemic had little to no effect on the thematic distribution within the discussions of the confident and hesitant communities. This aligns with the findings from Sobeczek, Gujski and Raciborski [61], who observed an intensification of the HPV vaccine discourse on Facebook during the COVID-19 vaccine distribution period but did not observe any changes in sentiment or theme of such online conversations. Therefore, public health professionals working today to craft HPV vaccine promotion messaging will not need to widely shift the focus on their messaging, as public discourse on the HPV vaccine does not appear to have shifted dramatically during the COVID-19 pandemic. Instead, public health professionals may wish to invest in raising awareness and interest in the HPV vaccine in general, given that public interest in COVID-19 and its vaccines have dominated public health conversations that previously included a focus on HPV vaccination. This is particularly important because research evidence from nations with previously high childhood vaccination rates, which include HPV vaccination, are seeing rates plummet in the wake of the COVID-19 pandemic [62–63].

Figure 4 shows the proportion of tweets mentioning COVID-19 within each community. As expected, there are no mentions of COVID-19 before early 2020. However, after the WHO declaration of a Global Health Emergency, both communities began mentioning COVID-19. These mentions trail off over summer and autumn of 2020, but in late December 2020, COVID-19 mentions pick up again in the HPV vaccine hesitant community. This corresponds with the earliest administrations of COVID-19 vaccines to vulnerable individuals. There are likely considerable numbers of individuals in the HPV vaccine hesitant community who are equally hesitant of the COVID-19 vaccines, and this development may have spurred this uptick in discussion within this community. It is also likely that increased awareness caused by COVID-19 vaccination drew attention to other vaccines less known to the public, accounting for this uptick in new accounts in the HPV vaccine hesitant community, as these individuals previously outside the HPV vaccine hesitant network drew comparisons between Gardasil and COVID-19 vaccines; which they were more familiar with.

Mentions among the HPV vaccine confident community did not increase significantly until April of 2021, when the COVID-19 vaccines began a much wider-scale rollout. It is likely that proponents of the HPV vaccine are also in support of the COVID-19 vaccines, and that this increase in discussion corresponds to a greater push for individuals to get vaccinated against COVID-19. While there is still a paucity of literature examining the impact of the COVID-19 pandemic on intention to receive the HPV vaccine, there is some evidence the COVID-19 pandemic has increased parental intention to vaccinate their children against the flu [64–65]. Thus, as high-income nations have begun to scale-back their COVID-19 vaccination campaigns, there is a need to re-introduce the value of the HPV vaccine.

Finally, in Fig. 5, we observed, first, a slight decrease of the unique vaccine confident Twitter accounts and an increase in unique vaccine hesitant Twitter accounts after the WHO declared COVID-19 as a Global Health Emergency of International Concern. It is likely that public health figures redirected their online attention to socializing individuals to comply with public health mandates and encouraging COVID-19 vaccine uptake. This likely diverted their attention from HPV vaccination and can account for the diminished vaccine confident activity over this period. Second, it seems that the COVID-19 pandemic represented an exogenous event that drew attention to other vaccines that were not previously in the public focus. Indeed, we see an increase by 40% of accounts associated with the HPV vaccine hesitant network during the COVID period. Furthermore, it seems that Twitter’s content moderation strategies did not significantly influence the dissemination of anti-vaccine content in the HPV vaccine hesitant network. On the one hand, we see a consistent growth in both vaccine hesitant narratives and engagement of new accounts in the vaccine hesitant network. On the other hand, a review of the top accounts in that space prior to and during COVID-19 suggest that such measures were limited. One would have expected Twitter to focus their content moderation efforts on the most prolific and influential individuals spreading anti-vaccine misinformation. Our assessment of the most influential accounts in the vaccine hesitant network reveals that Twitter only suspended the accounts of some of the least prominent influential figures and avoided taking on more powerful influencers and super spreaders engaged in a disinformation campaign on HPV vaccination.

## Strengths & limitations

To our knowledge, this is the first study using social network analysis and sentiment analysis to examine the impact of the COVID-19 pandemic on sentiments on HPV vaccination among English-language vaccine hesitant and vaccine confident networks on Twitter. The present study has several strengths. First, this study is reinforced by the undertaking of a rapid review of literature that informed the development of the HPV related keywords that were used to gather tweets and re-tweets used for analysis. Second, using network analysis and machine-learning text analysis allowed us to compare specific narratives and sentiment within vaccine hesitant and confident online conversations, thus providing a more nuanced understanding of the underlying frames relied on by these communities. Third, this study allowed us to assess the temporal evolution of discussion of HPV vaccination with reference to other epidemiological events (i.e., the COVID-19 pandemic).

There are several limitations in this study that could be addressed in future research. First, in terms of the time period of study, tweets were collected before the COVID-19 pandemic and during the start of the COVID-19 vaccine rollout. During this time, there was a COVID-19 vaccine eligibility requirement and not all individuals were qualified to be vaccinated. Therefore, the COVID-19 mentions in our dataset do not display the complete impact to online conversations and sentiment around the HPV vaccine amidst COVID-19 vaccine rollout. Further research is needed to see whether our findings are reflective of the entire COVID-19 pandemic, which as of writing is ongoing. Second, while Twitter is commonly used to study online social interactions, it does not represent the general population and particularly youth, who are the target population for HPV vaccination campaigns. For this reason, examining these research questions by also collecting data from other social media platforms such as Facebook, Reddit, Instagram, and YouTube, and analyzing HPV conversations from these platforms is an area for future study.

Finally, a fruitful area for further work is to investigate the impact of COVID-19 on the dynamics of the vaccine hesitant HPV network. While we have demonstrated the number of unique accounts in the vaccine hesitant HPV network grew over the course of the COVID-19 pandemic, future research should consider how the pandemic influenced the level of polarization or the level of interaction between the vaccine hesitant and vaccine confident networks. Further analysis of such dynamics could highlight whether the vaccine hesitant space has become more or less penetrable by information originating among vaccine confident users, including public health figures. While existing research has shown that increased polarization has resulted in different online contexts over the result of the COVID-19 pandemic [66], it remains to be seen if such polarization extended to online discussions about HPV immunization.

Further, while we have offered a limited analysis of the impact of Twitter’s content moderation policies on the dissemination of vaccine hesitant tweets related to HPV above, a full study of the impact of content moderation on anti-vaccine network structures is a rich and interesting topic deserving of its own paper. Such research would be highly topical, particularly given Elon Musk’s recent takeover of Twitter and ongoing debate around whether Twitter should serve as a “de facto public town square” [67]. An analysis on whether the misinformation environment on Twitter changed when moderation strategies were relaxed would meaningfully contribute to and enrich such debates.

## Conclusion

With the onset of the COVID-19 pandemic, discussions around the HPV vaccine on Twitter decreased among vaccine confident networks but we did not observe any significant changes in sentiment and themes surrounding the HPV vaccine during the COVID-19 pandemic and distribution of the COVID-19 vaccine. Safety concerns surrounding the HPV vaccine represent the most predominant theme discussed in the HPV vaccine hesitant networks. Further, the COVID-19 pandemic drew new users to engage in the HPV vaccine hesitant space. Therefore, as public health practitioners prioritize vaccine catch-up programs there is a need to raise public consciousness of the HPV vaccine and its benefits. The themes of such a campaign should prioritize a refutation of safety concerns, which is the primary critique of anti-Gardasil influencers.

## Data Availability

The data used in this study is publicly available on Twitter using an Academic Research Account (https://developer.twitter.com/en/products/twitter-api/academic-research).

## List of abbreviations

HPV: Human Papillomavirus
COVID-19: Coronavirus Disease 2019
STI: Sexually Transmitted Infection
WHO: World Health Organization
BERT: Bidirectional Encoder Representations from Transformers
TF-IDF: Term Frequency-Inverse Document Frequency
NLTK: Natural Language Toolkit
API: Application Programming Interface
SNA: Social Network Analysis
